# Supplementary material for: Long-Chain Polyunsaturated Fatty Acids Are Associated with Blood Pressure and Hypertension over 10-Years in Black South African Adults Undergoing Nutritional Transition
Source: Foods. 2019 Sep 6;8(9):394. doi: 10.3390/foods8090394 (PMC6770669; doi:10.3390/foods8090394)
Supplement: Supplementary file 1 [file foods-08-00394-s001.pdf]

Supplementary Tables

Supplementary Table S1. Blood pressure across 10 years in black South Africans.

	2005	2010	2015	*p* [1]
**Systolic blood pressure, mmHg**				
Complete sample, *n*	136.54 (23.29)	137.52 (24.87)	135.74 (25.56)	0.678
Rural areas, *n* 140	131.56 (22.81)	136.57 (26.92)	137.57 (27.12)	0.113
Urban areas, *n* 160	140.91 (22.89)	138.34 (23.02)	134.13 (24.07)	0.034
**Diastolic blood pressure, mmHg**				
Complete sample, *n*	89.04 (12.69)	88.55 (13.35)	85.37 (13.31)	0.001
Rural areas, *n* 140	87.10 (13.33)	87.92 (13.66)	87.31 (14.19)	0.877
Urban areas, *n* 160	90.75 (11.87)	89.09 (13.09)	83.66 (12.27)	<0.0001

Data presented as mean (SD). [1] Probability trends associated with differences across three study years calculated by general linear model.

Supplementary Table S2. Plasma phospholipids fatty acid status across 10-years in rural (n 140) and urban (n 160) black South Africans.

	2005		2010		2015			
	**Rural areas**	**Urban areas**	**Rural areas**	**Urban areas**	**Rural areas**	**Urban areas**	***p*** [1]	***p*** [2]
**Myristic acid, 14:0**	0.26 (0.00)	0.28 (0.00)	0.26 (0.00)	0.28 (0.00)	0.30 (0.01)	0.36 (0.01)	<0.0001	<0.0001
**Palmitic acid, 16:0**	26.62 (0.08)	27.21 (0.09)	27.63 (0.13)	26.77 (0.15)	25.47 (0.05)	24.20 (0.05)	0.002	<0.0001
**Palmitoleic acid, 16:1 n-7**	0.75 [0.71, 0.79]	0.96 [0.91, 1.00]	0.70 [0.67, 0.73]	0.91 [0.87, 0.94]	0.78 [0.74, 0.82]	1.03 [0.98, 1.08]	0.407	0.158
**Stearic acid, 18:0**	16.23 (0.30)	14.41 (0.34)	15.55 (0.15)	14.38 (0.17)	14.16 (0.06)	14.22 (0.07)	<0.0001	0.147
**Oleic acid, 18:1 n-9**	8.33 [8.17, 8.48]	9.26 [9.04, 9.43]	7.86 [7.69, 8.02]	8.93 [8.70, 9.12]	7.72 [7.61, 7.82]	8.62 [8.47, 8.74]	0.056	0.136
**Mead, 20:3 n-9**	0.25 [0.24, 0.25]	0.26 [0.25, 0.26]	0.25 [0.24, 0.26]	0.22 [0.22, 0.23]	0.19 [0.19, 0.20]	0.28 [0.27, 0.29]	0.001	0.451
**Linoleic acid, 18:2 n-6**	15.75 (0.30)	16.27 (0.34)	15.65 (0.21)	17.62 (0.23)	16.29 (0.17)	15.88 (0.20)	0.094	0.377
**γ-Linolenic, 18:3 n-6**	0.11 [0.11, 0.12]	0.12 [0.12, 0.13]	0.11 [0.11, 0.11]	0.13 [0.12, 0.13]	0.10 [0.09, 0.10]	0.11 [0.11, 0.12]	0.065	0.162
**Dihomo-γ-linolenic, 20:3**	2.96 (0.08)	2.87 (0.09)	2.87 (0.07)	2.91 (0.08)	3.29 (0.04)	3.65 (0.04)	<0.0001	<0.0001
**Arachidonic acid, 20:4 n-6**	13.83 (0.00)	13.35 (0.00)	14.98 (0.02)	14.37 (0.03)	18.48 (0.16)	17.82 (0.18)	<0.0001	<0.0001
**Adrenic, 22:4 n-6**	0.67 (0.00)	0.54 (0.00)	0.69 (0.00)	0.71 (0.00)	0.64 (0.01)	0.68 (0.01)	0.441	<0.0001
**Osbond, 22:5 n-6**	0.67 [0.67, 0.68]	0.56 [0.56, 0.57]	0.69 [0.69, 0.70]	0.73 [0.73, 0.74]	0.93 [0.92, 0.94]	1.09 [1.08, 1.10]	<0.0001	<0.0001
**α-linolenic acid, 18:3 n-3**	0.09 [0.09, 0.09]	0.09 [0.09, 0.10]	0.11 [0.11, 0.11]	0.09 [0.09, 0.09]	0.08 [0.07, 0.08]	0.07 [0.07, 0.08]	0.024	<0.0001
**EPA, 20:5 n-3**	0.59 [0.58, 0.60]	0.80 [0.79, 0.81]	0.60 [0.59, 0.61]	0.45 [0.44, 0.46]	0.52 [0.50, 0.55]	0.59 [0.55, 0.62]	0.070	<0.0001
**Docosapentaenoic, 22:5 n-**	1.39 (0.00)	1.43 (0.00)	1.62 (0.03)	1.24 (0.04)	1.61 (0.01)	1.46 (0.02)	<0.0001	0.366
**DHA, 22:6 n-3**	3.91 (0.06)	5.13 (0.07)	3.99 (0.04)	3.79 (0.04)	4.30 (0.13)	4.36 (0.15)	0.004	<0.0001

EPA, Eicosapentaenoic acid; DHA, Docosahexaenoic acid. Age-adjusted data presented as mean (SD) or median [25th, 75th]. [1] Probability trends associated with differences across three study years within rural subjects, calculated by general linear model adjusted for age. [2] Probability trends associated with differences across three study years within urban subjects, calculated by general linear model adjusted for age.

Supplementary Table S3. Baseline correlations between dietary intake of n-3 fatty acids and long-chain fatty acids in plasma phospholipids in 300 black South Africans: Pearson correlation matrix.

	**DGLA**	**AA**	**Adrenic**	**Osbond**	**EPA**	**DPA_n3**	**DHA**	**Intake_EPADHA**	**Intake_Omega 3**
**DGLA**	1	0.303<br><0.0001	0.413<br><0.0001	0.395<br><0.0001	0.012<br>0.833	0.208<br><0.0001	0.119<br>0.040	-0.091<br>0.117	-0.073<br>0.210
**AA**	0.303<br><0.0001	1	0.428<br><0.0001	0.444<br><0.0001	0.017<br>0.771	0.367<br><0.0001	0.392<br><0.0001	-0.136<br>0.018	-0.113<br>0.052
**Adrenic**	0.413<br><0.0001	0.428<br><0.0001	1	0.725<br><0.0001	-0.229<br><0.0001	0.221<br><0.0001	-0.243<br><0.0001	-0.126<br>0.030	-0.280<br><0.0001
**Osbond**	0.395<br><0.0001	0.444<br><0.0001	0.725<br><0.0001	1	-0.284<br><0.0001	-0.044<br>0.444	-0.054<br>0.355	-0.180<br>0.002	-0.198<br>0.001
**EPA**	0.012<br>0.833	0.017<br>0.771	-0.229<br><0.0001	-0.284<br><0.0001	1	0.454<br><0.0001	0.271<br><0.0001	0.096<br>0.095	0.138<br>0.017
**DPA_n3**	0.208<br><0.0001	0.367<br><0.0001	0.221<br><0.0001	-0.044<br>0.444	0.454<br><0.0001	1	0.352<br><0.0001	0.019<br>0.741	0.038<br>0.509
**DHA**	0.119<br>0.040	0.392<br><0.0001	-0.243<br><0.0001	-0.054<br>0.355	0.271<br><0.0001	0.352<br><0.0001	1	0.023<br>0.689	0.218<br><0.0001
**Intake_EPADHA**	-0.091<br>0.117	-0.136<br>0.018	-0.126<br>0.030	-0.180<br>0.002	0.096<br>0.095	0.019<br>0.741	0.023<br>0.689	1	0.769<br><0.0001
**Intake_Omega3**	-0.073<br>0.210	-0.113<br>0.052	-0.280<br><0.0001	-0.198<br>0.001	0.138<br>0.017	0.038<br>0.509	0.218<br><0.0001	0.769<br><0.0001	1

DGLA, dihommo-γ-linoleic acid; AA, arachidonic acid; EPA, Eicosapentaenoic acid; DPA_n3, docosapentaenoic acid; DHA, Docosahexaenoic acid; Intake_EPADHA, Cumulative intake of preformed EPA and DHA; Intake_Omega3, Cumulative intake of EPA, DHA and plant-originated essential α-linolenic acid.

Supplementary Table S4. Ten-year associations between plasma phospholipid long-chain fatty acids and blood pressure in rural and urban black South Africans.

	**Systolic blood pressure**				**Diastolic blood pressure**			
	**Rural subjects (*n* 140)**		**Urban subjects (*n* 160)**		**Rural subjects (*n* 140)**		**Urban subjects (*n* 160)**	
	β (95% CI)	*p* [3]	β (95% CI)	*p* [3]	β (95% CI)	*p* [3]	β (95% CI)	*p* [3]
**Long-chain n-3 fatty acids**								
**EPA, 20:5 n-3**								
T1	ref.		ref.		ref.		ref.	
T2[1]	1.09 (-3.82, 5.99)	0.867	1.69 (-2.71, 6.10)	0.247	1.64 (-1.19, 4.47)	0.504	1.15 (-1.36, 3.67)	0.276
T3	-0.07 (-5.08, 4.93)		3.75 (-0.65, 8.15)		1.24 (-1.65, 4.12)		2.05 (-0.46, 4.56)	
T2[2]	1.31 (-3.68, 6.30)	0.717	2.65 (-1.84, 7.14)	0.120	1.44 (-1.48, 4.36)	0.624	1.48 (-1.10, 4.07)	0.188
T3	-0.63 (-5.72, 4.46)		4.65 (0.21, 9.09)		0.79 (-2.18, 3.77)		2.36 (-0.20, 4.91)	
**Docosapentaenoic, 22:5 n-3**								
T1	ref.		ref.		ref.		ref.	
T2[1]	0.58 (-4.43, 5.59)	0.898	0.31 (-4.20, 4.82)	0.971	1.21 (-1.68, 4.09)	0.305	0.31 (-2.26, 2.87)	0.680
T3	-0.57 (-5.78, 4.63)		0.58 (-4.12, 5.28)		-1.01 (-4.01, 1.98)		-0.81 (-3.47, 1.86)	
T2[2]	-0.73 (-5.72, 4.27)	0.957	-1.44 (-5.97, 3.09)	0.780	0.36 (-2.55, 3.28)	0.566	-0.46 (-3.05, 2.14)	0.738
T3	-0.58 (-5.84, 4.68)		-0.09 (-4.81, 4.62)		-1.19 (-4.25, 1.87)		-1.07 (-3.76, 1.63)	
**DHA, 22:6 n-3**								
T1	ref.		ref.		ref.		ref.	
T2[1]	-1.37 (-6.35, 3.61)	0.174	-0.75 (-5.34, 3.83)	0.943	-1.68 (-4.56, 1.19)	0.132	-1.31 (-3.92, 1.30)	0.588
T3	-4.91 (-10.28, 0.47)		-0.66 (-5.44, 4.12)		-3.18 (-6.27, -0.08)		-1.10 (-3.81, 1.62)	
T2[2]	-2.14 (-7.14, 2.86)	0.207	-0.45 (-5.06, 4.16)	0.982	-2.33 (-5.25, 0.58)	0.049	-1.07 (-3.71, 1.57)	0.719
T3	-4.87 (-10.27, 0.54)		-0.26 (-5.16, 4.64)		-3.91 (-7.04, -0.78)		-0.42 (-3.22, 2.38)	
**Long-chain n-6 fatty acids**								
**Dihomo-γ-linolenic acid, 20:3 n-6**								
T1	ref.		ref.		ref.		ref.	
T2[1]	4.09 (-0.90, 9.07)	0.253	-0.57 (-5.02, 3.88)	0.179	1.13 (-1.75, 4.00)	0.331	-0.23 (-2.75, 2.29)	0.012
T3	3.09 (-1.93, 8.12)		-3.99 (-8.60, 0.61)		2.20 (-0.71, 5.10)		-3.51 (-6.11, -0.91)	

T2[2]	1.89 (-3.14, 6.92)	0.760	-0.57 (-5.10, 3.96)	0.173	0.06 (-2.88, 2.99)	0.824	0.05 (-2.53, 2.64)	0.020
T3	0.94 (-4.10, 5.98)		-4.19 (-8.99, 0.62)		0.81 (-2.13, 3.75)		-3.29 (-6.03, -0.55)	
**Arachidonic acid, 20:4 n-6**								
T1	ref.		ref.		ref.		ref.	
T2[1]	3.80 (-0.91, 8.52)	0.273	-2.49 (-6.83, 1.85)	0.001	1.60 (-1.14, 4.33)	0.456	-2.20 (-4.60, 0.21)	<0.0001
T3	2.61 (-2.16, 7.38)		-8.16 (-12.46, -3.86)		0.22 (-2.55, 2.98)		-7.01 (-9.39, -4.62)	
T2[2]	3.32 (-1.42, 8.07)	0.383	-2.43 (-6.79, 1.93)	0.003	1.79 (-1.00, 4.58)	0.298	-2.16 (-4.59, 0.28)	<0.0001
T3	1.38 (-3.41, 6.16)		-7.57 (-11.97, -3.18)		-0.21 (-3.02, 2.60)		-6.74 (-9.20, -4.29)	
**Adrenic acid, 22:4 n-6**								
T1	ref.		ref.		ref.		ref.	
T2[1]	-1.07 (-5.98, 3.83)	0.194	-2.61 (-6.99, 1.76)	0.467	1.20 (-1.63, 4.03)	0.200	-0.79 (-3.28, 1.70)	0.470
T3	3.33 (-1.93, 8.59)		-0.64 (-5.28, 3.99)		2.76 (-0.27, 5.78)		-1.65 (-4.28, 0.98)	
T2[2]	-2.02 (-6.98, 2.94)	0.281	-3.00 (-7.34, 1.35)	0.383	0.63 (-2.26, 3.53)	0.247	-0.62 (-3.11, 1.87)	0.547
T3	1.94 (-3.43, 7.32)		-1.07 (-5.79, 3.65)		2.52 (-0.60, 5.63)		-1.51 (-4.20, 1.19)	
**Osbond acid, 22:5 n-6**								
T1	ref.		ref.		ref.		ref.	
T2[1]	-1.51 (-6.27, 3.24)	0.126	-2.85 (-7.16, 1.47)	0.071	-1.31 (-4.08, 1.45)	0.463	-1.98 (-4.39, 0.43)	<0.0001
T3	3.67 (-1.67, 9.00)		-5.37 (-9.94, -0.79)		0.35 (-2.72, 3.43)		-5.56 (-8.11, -3.00)	
T2[2]	-1.34 (-6.16, 3.49)	0.597	-3.54 (-7.84, 0.75)	0.046	-0.92 (-3.75, 1.91)	0.788	-2.28 (-4.71, 0.14)	<0.0001
T3	1.28 (-4.20, 6.76)		-5.83 (-10.50, -1.17)		-0.17 (-3.34, 3.01)		-5.55 (-8.17, -2.92)	

EPA, Eicosapentaenoic acid; DHA, Docosahexaenoic acid; T1, T2, T3, Increasing tertiles of plasma phospholipid fatty acid content.

[1]Model 1 adjusted for age and gender. [2]Model 2 further adjusted for level of education, use of tobacco, use of hypertension medication, body mass index, physical activity index and dietary intake of alcohol (g). [3] Probability values associated with β estimating absolute change in blood pressure (in mmHg) with regards to 10-year change in a fatty acid level.

Supplementary Table S5. Ten-year associations between plasma phospholipid long-chain fatty acids and hypertension status in black South Africans.

	**General (*n* 300)**		**Rural subjects (*n* 140)**		**Urban subjects (*n* 160)**	
	OR (95% CI)	p^3	OR (95% CI)	p^3	OR (95% CI)	p^3
**Long-chain n-3 fatty acids**						
**EPA, 20:5 n-3**						
T1	ref.		ref.		ref.	
T2[1]	1.43 (1.03, 1.99)	0.093	1.39 (0.87, 2.21)	0.190	1.27 (0.79, 2.05)	0.589
T3	1.28 (0.92, 1.78)		1.53 (0.95, 2.47)		1.07 (0.68, 1.70)	
T2[2]	1.57 (1.10, 2.24)	0.028	1.46 (0.86, 2.49)	0.134	1.46 (0.88, 2.40)	0.341
T3	1.46 (1.03, 2.08)		1.72 (1.00, 2.98)		1.19 (0.73, 1.93)	
**Docosapentaenoic acid, 22:5 n-3**						
T1	ref.		ref.		ref.	
T2[1]	1.16 (0.83, 1.62)	0.594	1.14 (0.71, 1.83)	0.300	1.05 (0.65, 1.69)	0.714
T3	1.00 (0.71, 1.41)		1.47 (0.90, 2.40)		0.87 (0.54, 1.40)	
T2[2]	1.15 (0.80, 1.64)	0.700	1.09 (0.64, 1.85)	0.330	1.02 (0.62, 1.68)	0.810
T3	1.01 (0.70, 1.46)		1.50 (0.86, 2.62)		0.88 (0.53, 1.45)	
**DHA, 22:6 n-3**						
T1	ref.		ref.		ref.	
T2[1]	1.02 (0.73, 1.42)	0.840	1.11 (0.69, 1.78)	0.436	0.72 (0.45, 1.17)	0.370
T3	1.10 (0.78, 1.57)		1.38 (0.83, 2.30)		0.92 (0.56, 1.52)	
T2[2]	1.01 (0.70, 1.44)	0.994	0.99 (0.57, 1.70)	0.658	0.73 (0.44, 1.20)	0.459
T3	1.02 (0.70, 1.50)		1.25 (0.70, 2.23)		0.86 (0.51, 1.47)	
**Long-chain n-6 fatty acids**						
**Dihomo-γ-linolenic acid, 20:3 n-6**						
T1	ref.		ref.		ref.	
T2[1]	1.47 (1.06, 2.04)	0.038	1.95 (1.22, 3.13)	0.001	1.08 (0.67, 1.74)	0.587
T3	1.44 (1.03, 2.00)		2.43 (1.50, 3.94)		0.85 (0.53, 1.37)	
T2[2]	1.38 (0.97, 1.97)	0.156	1.62 (0.95, 2.79)	0.042	1.08 (0.66, 1.78)	0.678
T3	1.33 (0.93, 1.92)		1.99 (1.15, 3.43)		0.86 (0.51, 1.45)	
**Arachidonic acid, 20:4 n-6**						
T1	ref.		ref.		ref.	
T2[1]	0.83 (0.60, 1.15)	0.536	1.48 (0.94, 2.34)	0.049	0.67 (0.41, 1.07)	0.150
T3	0.91 (0.65, 1.26)		1.75 (1.11, 2.78)		0.66 (0.41, 1.06)	
T2[2]	0.82 (0.58, 1.17)	0.512	1.42 (0.84, 2.40)	0.147	0.67 (0.41, 1.10)	0.131
T3	0.85 (0.60, 1.21)		1.69 (0.99, 2.88)		0.62 (0.37, 1.01)	
**Adrenic acid, 22:4 n-6**						
T1	ref.		ref.		ref.	
T2[1]	1.13 (0.82, 1.57)	0.757	1.09 (0.68, 1.75)	0.878	1.04 (0.66, 1.65)	0.907
T3	1.08 (0.77, 1.52)		1.13 (0.69, 1.86)		1.11 (0.69, 1.80)	
T2[2]	1.16 (0.82, 1.64)	0.701	1.12 (0.65, 1.91)	0.913	1.08 (0.67, 1.75)	0.836
T3	1.11 (0.77, 1.61)		1.10 (0.63, 1.94)		1.17 (0.70, 1.95)	
**Osbond acid, 22:5 n-6**						

T1	ref.		ref.		ref.	
T2[1]	1.25 (0.91, 1.73)	0.372	1.54 (0.98, 2.45)	0.005	0.93 (0.58, 1.49)	0.372
T3	1.19 (0.85, 1.67)		2.35 (1.41, 3.91)		0.72 (0.45, 1.17)	
T2[2]	1.23 (0.87, 1.75)	0.481	1.52 (0.89, 2.59)	0.030	0.95 (0.58, 1.54)	0.458
T3	1.16 (0.81, 1.67)		2.22 (1.23, 4.02)		0.74 (0.45, 1.23)	

EPA, Eicosapentaenoic acid; DHA, Docosahexaenoic acid; T1, T2, T3, Increasing tertiles of plasma phospholipid fatty acid content. [1] Model 1 adjusted for age, gender and urbanization status (the latter in the complete sample only). [2] Model 2 further adjusted for level of education, use of tobacco, body mass index, physical activity index and dietary intake of alcohol (g). [3] Probability values associated with OR of being hypertensive across the 10-years.

Supplementary Figures

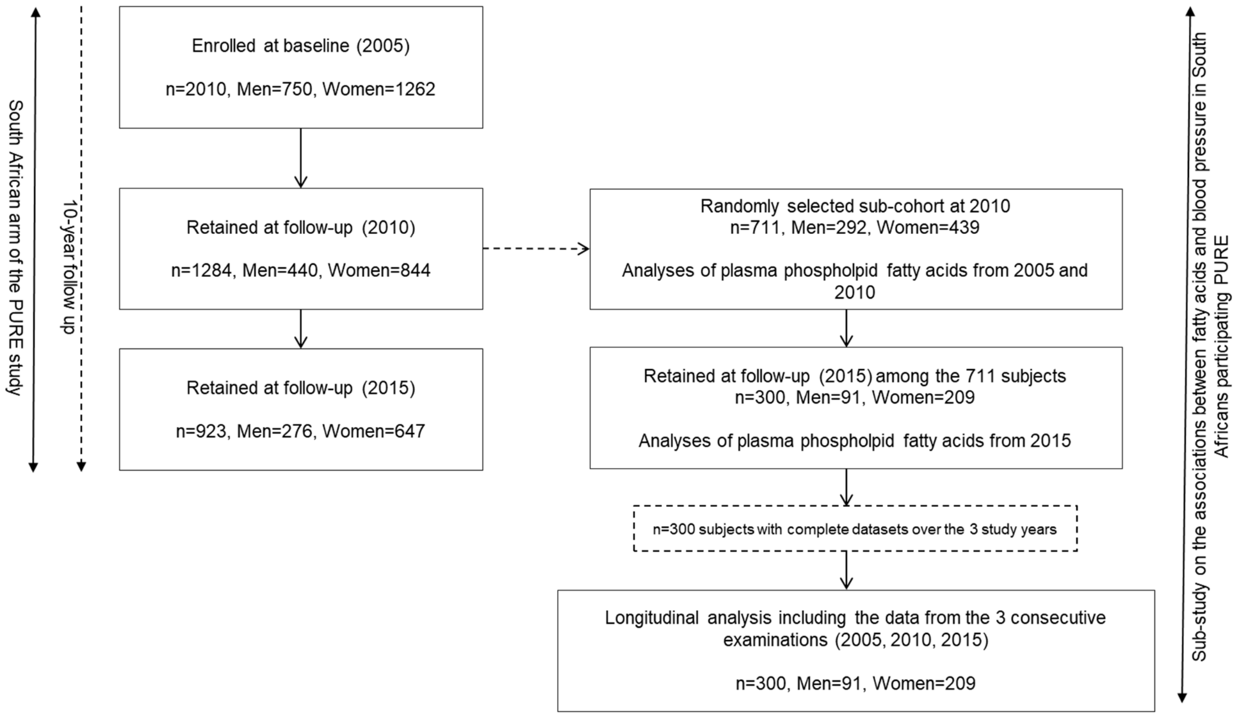

Supplementary Figure S1. Flow-diagram explaining selection of the 300 black South Africans for the longitudinal analysis of associations between fatty acids and blood pressure over 10 years.
